# Densification and Proton Conductivity of La_1-x_Ba_x_ScO_3-δ_ Electrolyte Membranes

**DOI:** 10.3390/membranes12111084

**Published:** 2022-10-31

**Authors:** Alyona Lesnichyova, Semyon Belyakov, Anna Stroeva, Sofia Petrova, Vasiliy Kaichev, Anton Kuzmin

**Affiliations:** 1 Laboratory of Solid-State Chemistry, Institute of Solid-State Chemistry and Mechanochemistry SB RAS, 630128 Novosibirsk, Russia; 2 NANOTECH Centre, Ural Federal University, 620075 Yekaterinburg, Russia; 3 Laboratory of Electrochemical Materials Science, Institute of High-Temperature Electrochemistry UB RAS, 620137 Yekaterinburg, Russia; 4 Department of Technology of Inorganic Materials and Electrochemical Production, Vyatka State University, 610000 Kirov, Russia; 5 Laboratory of High-Entropy Alloys, Institute of Metallurgy UB RAS, 620016 Yekaterinburg, Russia; 6 Department of Catalysis Research, Boreskov Institute of Catalysis SB RAS, 630090 Novosibirsk, Russia

**Keywords:** proton conductivity, lanthanum scandate, sintering additive

## Abstract

Bain La_1-x_Ba_x_ScO_3-δ_ impairs sintering and leads to a decrease in its ceramic density. Two approaches have been studied for obtaining dense ceramics: using a high processing temperature and the introduction of a Co_3_O_4_ sintering additive. An addition of only 0.5 wt% of Co_3_O_4_ sintering additive, despite the positive sintering effect, causes a noticeable violation of stoichiometry, with partial decomposition of the material. This can lead to the formation of cationic vacancies, which form associates with oxygen vacancies and significantly reduce the oxygen ion and proton conductivity of the materials. There is also a partial substitution of Co for Sc in La_1-x_Ba_x_ScO_3-δ_, which reduces the stability of protons: it reduces the enthalpy of the hydration reaction, but increases the mobility of protons. Thus, the Co_3_O_4_ sintering additive causes a complex of negative effects on the conductivity of La_1-x_Ba_x_ScO_3-δ_ materials. Only high-temperature (1800 °C) processing with protection against Ba loss contributes to the production of dense La_1-x_Ba_x_ScO_3-δ_ ceramics. The chemical composition of such ceramics corresponds well to the specified one, which ensures high water uptake and, consequently, high proton conductivity.

## 1. Introduction

Materials with proton conductivity are currently one of the most interesting among solid oxide materials. Among proton-conducting oxides, the highest proton conductivity is realized in acceptor-doped materials with a perovskite-type structure, which makes them promising for use in electrochemical devices, for example, as solid electrolytes for proton–ceramic fuel cells, gas sensors, etc. [1,2,3,4,5,6,7,8]. Their feature is that proton charge carriers are formed by incorporating water vapor into oxygen vacancies, formed, for example, by introducing a cationic acceptor dopant with a reduced valence:

(1) $$\mathrm{BaO}\left(-{\mathrm{LaO}}_{1.5}\right)\to {\mathrm{Ba}}_{\mathrm{La}}^{/}+\frac{1}{2}V_{O}^{••}+O_{O}^{\times },$$

(2) $$H_{2}O+V_{O}^{••}+O_{O}^{\times }=2{\mathrm{OH}}_{O}^{•},$$

where ${\mathrm{Ba}}_{\mathrm{La}}^{/}$ are Ba dopant atoms in La regular lattice positions, $O_{O}^{\times }$ are oxygen atoms in regular lattice positions, $V_{O}^{••}$ are oxygen vacancies, and ${\mathrm{OH}}_{O}^{•}$ are proton defects.

The perovskite group of the A^3+^B^3+^O_3_ type, such as La_1-x_M_x_BO_3-δ_ (where M = Ca, Sr, Ba; B = Y, Yb, Sc, In) [8,9,10,11,12,13,14,15,16,17,18,19,20,21,22,23,24,25,26,27], are promising ceramic proton conductors because they have a significant advantage over the widely studied materials based on Ba(Zr,Ce)O_3_ due to the tendency of Ba to carbonize [28,29,30]. Systematic studies of the transport properties of La_1-x_M_x_BO_3-δ_ demonstrate a tendency for an increase in proton conductivity with an increase in the ionic radius of the dopant and a decrease in the ionic radius of the B-cation [8,9,10,11,12,13,14,15,16,17,18,19,20,21,22,23,24,25,26,27]; thus, the highest proton conductivity should be realized in La_1-x_Ba_x_ScO_3-δ_.

Unfortunately, there is no information on La_1-x_Ba_x_ScO_3-δ_ ceramic sintering in the literature. Lee et al. [15] do not provide information on the density of La_1-x_Ba_x_ScO_3-δ_ (x = 0.05–0.50) ceramics sintered at 1600 °C (10 h), despite the fact that the crystal structure and conductivity of these materials have been studied in detail in a wide range of Ba concentrations. They showed a change in the crystal structure when the concentration of Ba increases, namely: at 0 ≤ x < 0.1, an orthorhombic structure is formed, at 0.1 ≤ x < 0.3, a mixture of orthorhombic and cubic is formed, and at 0.3 ≤ x ≤ 0.5, only a cubic structure is presented. Kendrick et al. [31] carried out multiple anneals at 1500 °C (10 h) to prepare single-phase La_0.6_Ba_0.4_ScO_3-δ_, but obtained an actual Ba content of 0.27 and an orthorhombic structure, as well as an impurity of Sc_2_O_3_, which is detectable only due to the neutron diffraction technique.

On the example of La_1-x_Ba_x_YbO_3-δ_, it was shown that even the use of a small amount of Ba can complicate the sintering of ceramics. Kasyanova et al. [26] showed that the sintering of La_1-x_Ba_x_YbO_3-δ_ (x = 0.03–0.1) ceramics significantly deteriorates at 1400 °C (5 h) at a concentration of Ba x ≥ 0.05. On the other hand, Obukuro et al. [27] demonstrated the production of high-density ceramics (more than 95%) of La_1-x_Ba_x_YbO_3-δ_ (x = 0.02–0.1) after multistage synthesis and long-term sintering (10 h) at 1700 °C, although the authors did not check the Ba losses.

An alternative way to improve the sinterability of ceramics can be the introduction of small amounts of sintering additives, most often, transition metal oxides, into the pre-synthesized powder [32,33]. However, the sintering additive can negatively affect the water uptake and ionic conductivity of Ba-doped LaScO_3_, as shown in recent studies for Ba(Zr,Ce,Y)O_3_ materials [34,35,36,37]. The incorporation of over-stoichiometric transition metal atoms into the crystal lattice of proton-conducting materials generally has a negative effect on hydration, in particular, by reducing the saturation limit of protons. According to a study by Han et al. [33], oxides of Co and Ni in the Mn-Fe-Co-Ni series have the least negative effect on the proton transport of Ba(Zr,Y)O_3_ materials. We have previously identified Co_3_O_4_ among transition metal oxides as the optimal sintering additive for La_0.9_Sr_0.1_ScO_3-δ_ [8], while the effect of the same additive on La_1-x_Ba_x_ScO_3-δ_ has not been studied.

In this research, we investigated the effect of heat treatment regimes, as well as 0.5 wt% Co_3_O_4_, on the sintering process and microstructure, crystal structure, water uptake, and proton conductivity of the La_1-x_Ba_x_ScO_3-δ_ proton-conducting materials.

## 2. Materials and Methods

Materials of composition La_1-x_Ba_x_ScO_3-δ_ (where x = 0.025, 0.05, 0.075, and 0.1) were synthesized using the citrate–nitrate combustion method. Oxides La_2_O_3_ and Sc_2_O_3_, and carbonate BaCO_3_ (all of high pure grade), were used as precursors. Stoichiometric amounts of precursors, taking into account their weight loss coefficients during calcination, were converted to a nitrate solution using a nitric acid. After complete dissolution of all precursors, citric acid was added to the resulting mixture and evaporated until the combustion reaction proceeded. The powders obtained after drying were annealed at a temperature of 800 °C for 2 h for decarbonization, after which the powder mixture was homogenized in isopropyl alcohol using a PM 100 zirconia-based planetary ball mill (Retch GmbH, Haan, Germany) for 1 h (350 rpm). Dried powders of all compositions were subjected to preliminary synthesis at 1200 °C for 2 h. Parallel to the above path, 0.5 wt% Co_3_O_4_ (high pure grade) was added above the basic stoichiometry to the La_0.95_Ba_0.05_ScO_3-δ_ powder obtained after annealing at 800 °C, after which this mixture was homogenized in the planetary ball mill for 1 h (350 rpm) and calcined in air at 1200 °C for 2 h.

From the powders obtained by two above methods, compacts were made using a uniaxial hydraulic press at 400 Pa, which were then covered in the sacrificial powder of the same composition and sintered in air at 1650 °C for 5 h. In the case of sample La_0.95_Ba_0.05_ScO_3-δ_ obtained after two preliminary sintering at 1400 °C (1 h) and 1650 °C (5 h), additional sintering was carried out in a vacuum furnace at 1800 °C for 2 h using a sacrificial powder of the appropriate composition. Then, the obtained ceramic samples were calcined in air at 1100 °C for 5 h for oxygen stoichiometry relaxation. The shrinkage of compacts during heat treatment in air was studied by optical dilatometry using an ODP-868 platform (TA Instruments, New Castle, DE, USA) in the temperature range from room temperature to 1575 °C.

The identification of phases, as well as the crystal structure, was carried out using powder X-ray diffraction (XRD) analysis on a D/MAX-2200 diffractometer (Rigaku, Tokyo, Japan). High-temperature XRD analysis was performed in dry air on a D8 Advance diffractometer (Bruker, Karlsruhe, Germany) with a XRK-900 high-temperature chamber (Anton Paar, Graz, Austria). In both cases, the Cu Kα radiation was used. A dry atmosphere (*p*H_2_O = 0.1 kPa) was achieved by gas circulation through a column with zeolites. The measurements were carried out in a temperature range from room temperature to 900 °C, in the heating mode with a step of 20 °C and in the cooling mode with a step of 30 °C and with an exposure time of 8 min at each point. Phase analysis was performed using the DIFFRAC^plus^:EVA software (Bruker, Karlsruhe, Germany) [38] and PDF4 + ICDD database (Release 2021) [39]. The unit cell parameters were calculated using the Celref software (ENSP, Grenoble, France) [40]. Rietveld structure refinement was performed using the DIFFRAC^plus^: TOPAS software (Bruker, Karlsruhe, Germany) [41].

The cross-sections and surface of the ceramic samples were investigated by scanning electron microscopy (SEM) using MIRA 3 LMU equipment (Tescan, Brno, Czech Republic). The elemental distribution was studied by energy-dispersive X-ray (EDX) spectroscopy using INCA Energy 350 X-Max 80 equipment (Oxford Instruments, Concord, MA, USA).

X-ray photoelectron spectroscopy (XPS) analysis of the samples was carried out on a X-ray photoelectron spectrometer equipped with a hemispherical analyzer PHOIBOS-150-MCD-9 and XR-50 source of X-ray radiation (SPECS Surface Nano Analysis GmbH, Berlin, Germany). The spectra were recorded using nonmonochromatized Al Kα radiation (1486.6 eV). The charge correction was performed by setting the Sc*2p* peak at 402.0 eV. The background was subtracted by the Shirley method. Quantitative analysis was performed using the integrated intensities of core-level spectra with the cross-sections according to Scofield [42]. The curve fitting was completed with CasaXPS software (Casa Software Ltd., Teignmouth, UK) [43]. The shape of the peaks is approximated by a symmetric function obtained by multiplying the Gauss and Lorentz functions.

The thermal expansion of the dense ceramic bars was studied using a quartz dilatometer and Tesatronic TT-80 equipment (TESA, Renens, Switzerland) from room temperature to 900 °C with a heating/cooling rate of 2 °C min^−1^ in dry air (*p*H_2_O = 0.1 kPa).

The thermogravimetric analysis (TGA) was performed on the powder samples with a specific surface area of 1.1 ± 0.2 m^2^/g using a STA Jupiter 449 F3 analyzer (Netzsch, Selb, Germany) with an aSTEAM DV2MK (aDROP Feuchtemeßtechnik GmbH, Fürth, Germany) water vapor generator. The as-prepared powders were heated up to 950 °C and held at this temperature for 8 h under the dry argon. Afterwards, the carrier gas was saturated with water vapor (*p*H_2_O = 24.3 kPa), and then the increase in weight was recorded upon cooling from 950 °C to 300 °C with a cooling rate of 30 °C h^−1^ and exposure every 100 °C over 2 h. The proton concentration was obtained from the weight change during TGA as follows:

(3) $$\Delta n_{\mathrm{OH}}=\frac{2\cdot \Delta m\cdot M_{s}}{m_{s}\cdot M_{H2O}}\;,$$

where M_s_ and M_H2O_ are molecular weights of the oxides and water, and Δm and m_s_ are the weight change and weight of the specimen, respectively.

The total conductivity was measured as a function of the temperature (*T* = 900–400 °C), oxygen partial pressure (*p*O_2_ = 21.3 kPa–10^−15^ Pa), and water partial pressure (*p*H_2_O = 0.1 and 2.8 kPa) by the four-probe DC method using an ADAM-3000 instrument (Advantech, Taiwan). The samples were rectangular bars with four Pt electrodes, which were made of dispersed Pt powder and sintered at 1100 °C during 1 h. The *p*O_2_ was controlled by an electrochemical oxygen pump and a sensor based on an yttria-stabilized zirconia (YSZ). The partial conductivities of protons, oxygen ions, and holes were calculated on the basis of the dependences of the total conductivity on *p*O_2_ and *p*H_2_O in the framework of the approach developed by Frade [44] and Baek [45]. A detailed description of the calculations is given in [24]. The experimental dependences of total conductivity on (*p*O_2_)^1/4^, which were obtained at two humidity values 2.8 kPa and 0.1 kPa, were used for the calculations and are given in the Supplementary Materials (Figure S1).

The electrochemical impedance spectroscopy (EIS) was performed at 450–300 °C in wet air using a SP-200 m (Bio-Logic, Seyssinet-Pariset, France). The measurements were carried out in a frequency range of 7 MHz–500 mHz and at a sinus signal amplitude of 100 mV. The impedance spectra were interpreted into bulk and grain boundary (GB) components using an equivalent circuit scheme of R_0_–(R_1_Q_1_)–(R_2_Q_2_), where R is the resistance, Q is the constant phase element, and the indexes of 1 and 2 correspond to the bulk and GB processes, respectively. R_0_ was purposefully introduced in the equivalent circuit scheme; it imitates the origin of the coordinates, providing a correct fitting. In the approximation of equality of the dielectric constants of the bulk and GBs [46], the GB resistance can be converted to a specific value using the formula:

(4) $$\sigma_{\mathrm{gb}}^{*}=\frac{1}{R_{\mathrm{gb}}}\cdot \frac{l}{S}\cdot \frac{C_{\mathrm{bulk}}}{C_{\mathrm{gb}}}\;,$$

where $R_{\mathrm{gb}}$ is the GB resistance, $\frac{l}{S}$ is the geometric parameter, and $C_{\mathrm{bulk}}$ and $C_{\mathrm{gb}}$ are the capacities of the bulk and GB, respectively.

## 3. Results

### 3.1. Materials Characterization

**Figure 1:**
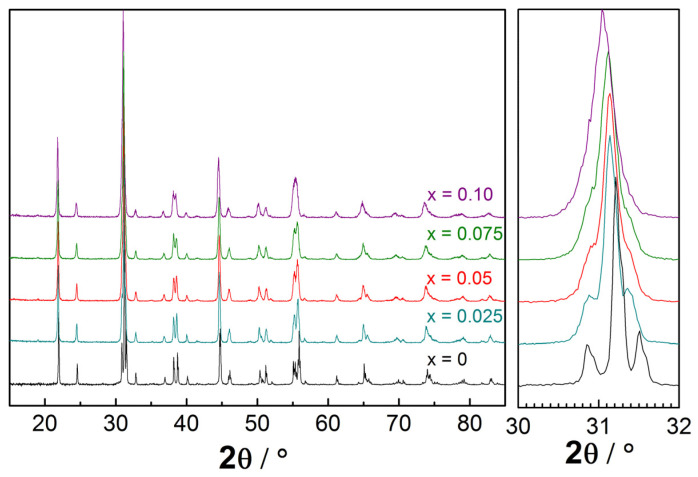
XRD patterns of La_1-x_Ba_x_ScO_3-δ_ samples.

**Figure 2:**
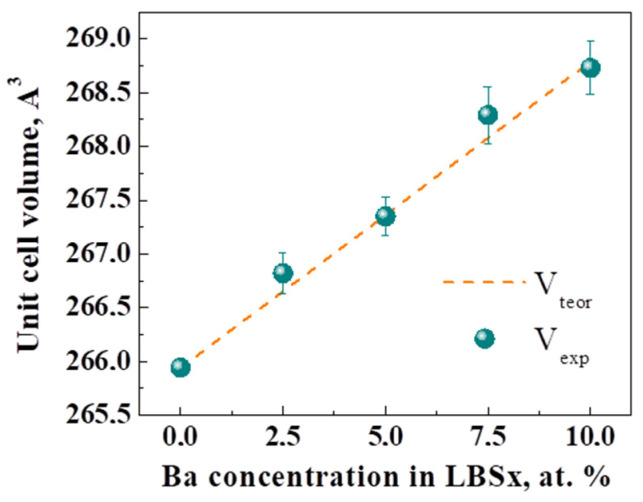
Unit cell volume of La_1-x_Ba_x_ScO_3-δ_ samples.

Figure 1 demonstrates the XRD patterns of prepared and dried powders of La_1-x_Ba_x_ScO_3-δ_ (0–0.1) obtained by combustion synthesis with final calcination at 1650 °C. The crystal structure of La_1-x_Ba_x_ScO_3-δ_ was characterized as an orthorhombic perovskite with the *Pnma* space group. No impurity phases were observed, which corresponds to a wide range of Ba dissolution in LaScO_3_ [15]. An increase in the Ba concentration leads to the gradual displacement of Bragg peaks; thus, the unit cell volume of La_1-x_Ba_x_ScO_3-δ_ increases (Figure 2). This is in a good agreement with the conventional representation of ionic radii by Shannon [47] because the ionic radius of Ba (rBa^2+^ = 1.61 Å) is larger than La (rLa^3+^ = 1.36 Å). Thus, when Ba replaces La, the crystal lattice of La_1-x_Ba_x_ScO_3-δ_ undergoes expansion [48] according to the equations:

(5) $$V=4\cdot a_{p}^{3}\;,$$

(6) $$a_{p}=\frac{A}{\surd 2}\cdot \left(\left(1-x)\cdot r_{\mathrm{La}}+x\cdot r_{\mathrm{Ba}}\right)+r_{O}\right)+B\cdot \left(r_{\mathrm{Sc}}+r_{O}\right)+C\;,$$

where $a_{p}$ is the pseudocubic unit cell parameter; A, B, and C are empirical parameters equal to 0.816, 1.437, and −0.63095, respectively [48]; and $\;r_{\mathrm{La}},{\;r}_{\mathrm{Ba}},{\;r}_{\mathrm{Sc}},{\;r}_{O}$ are Shannon ionic radii of La^3+^, Ba^2+^, Sc^3+^, and O^2−^ [47], respectively. In Figure 2 we can see that the theoretically calculated values of the unit cell volume are in good agreement with the values experimentally determined by XRD. It is important to note that we deliberately dried the samples prior to the XRD analysis in order to prevent additional hydration-related materials expansion.

**Figure 3:**
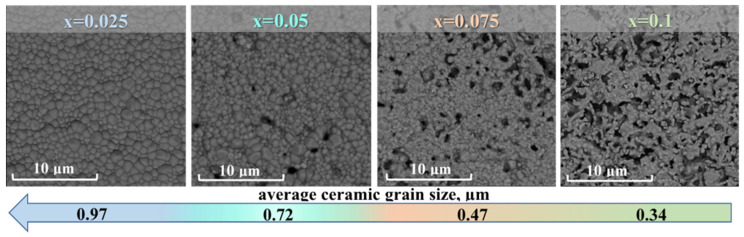
SEM images of surface morphology of La_1-x_Ba_x_ScO_3-δ_ ceramics.

According to Figure 3, the relative density of sintered La_1-x_Ba_x_ScO_3-δ_ ceramic materials decreases from ~94% for x = 0.025 to ~76% for x = 0.1. The average grain size also decreases with increases in the Ba concentration. Both trends show the deterioration of the sintering of ceramics based on LaScO_3_ caused by the presence of Ba, although similar trends were not previously observed for Ca- and Sr-doped LaScO_3_ [20,21,24]. Thus, obtaining dense La_1-x_Ba_x_ScO_3-δ_ ceramic materials is a difficult task, and therefore, taking La_0.95_Ba_0.05_ScO_3-δ_ as an example, we considered various strategies for producing dense ceramics.

### 3.2. Dense Ceramic Formation Strategies

**Figure 4:**
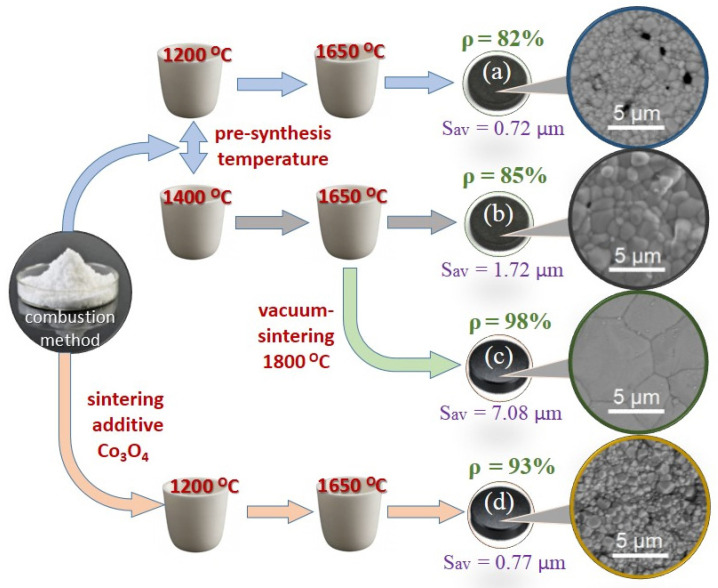
Sintering scheme for La_0.95_Ba_0.05_ScO_3-δ_ ceramics. ρ is the relative density, and S_av_ is the average grain size. The explanation for subfigures are in the text of the paper.

**Figure 5:**
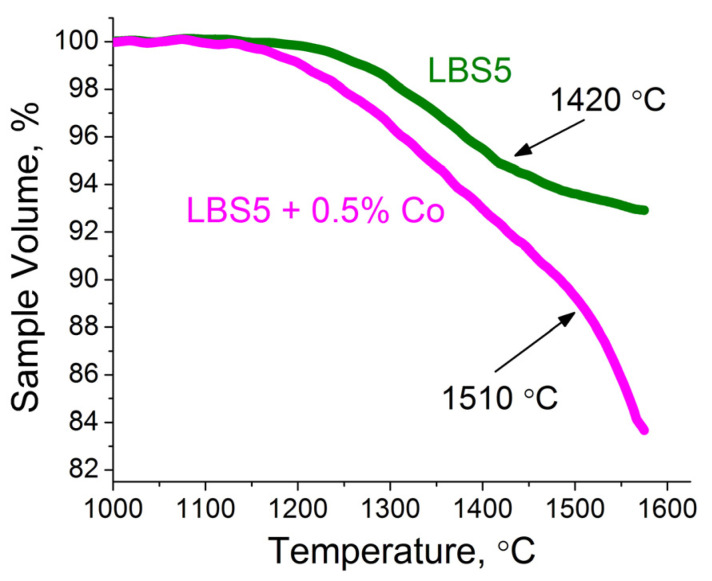
Shrinkage of La_0.95_Ba_0.05_ScO_3-δ_ and La_0.95_Ba_0.05_ScO_3-δ_ + 0.5 wt% Co_3_O_4_ samples during sintering according to optical dilatometry analysis.

The general scheme for studying the sintering process of La_0.95_Ba_0.05_ScO_3-δ_ samples is shown in Figure 4. As you can see in Figure 5, the most intense stage of shrinkage of La_0.95_Ba_0.05_ScO_3-δ_ occurs up to a temperature of 1420 °C and is only 6% of the initial compact volume. With a further increase in temperature, the shrinkage rate decreases, since the process of grain growth begins to compete with the shrinkage of the material, which leads to the formation of porous ceramics with a relative density of 82% after sintering at 1650 °C over 5 h (Figure 4a).

Based on the temperature dependence of shrinkage for La_0.95_Ba_0.05_ScO_3-δ_ samples (Figure 5), the pre-sintering temperature was increased from 1200 to 1400 °C, which led to an increase in the average grain size of the ceramic to 1.72 ± 0.42 μm, while the relative density still does not exceed 85% after 1650 °C sintering over 5 h (Figure 4b). This result prompted us to carry out additional sintering in a vacuum furnace, where the temperature reached 1800 °C after 2 h. As can be seen from Figure 4c, after vacuum sintering, it was possible to obtain ceramics with a relative density of 98% and an impressive average grain size of 7.08 ± 1.19 μm.

We introduced 0.5 wt% Co_3_O_4_ into the synthesized La_0.95_Ba_0.05_ScO_3-δ_ powder, since it was shown earlier [8] that cobalt oxide is the optimal additive for La_0.9_Sr_0.1_ScO_3-δ_. As Figure 5 shows, the densification process becomes more active with the addition of Co_3_O_4_: it begins at 1150 °C and reaches 12% at 1510 °C, and with a further increase in temperature, the sintering rate increases. In this case, the process of grain splicing dominates, which leads to an increase in the density of ceramics up to ~93% after sintering at 1650 °C over 5 h (Figure 4d).

### 3.3. Dense Ceramics Characterization

**Figure 6:**
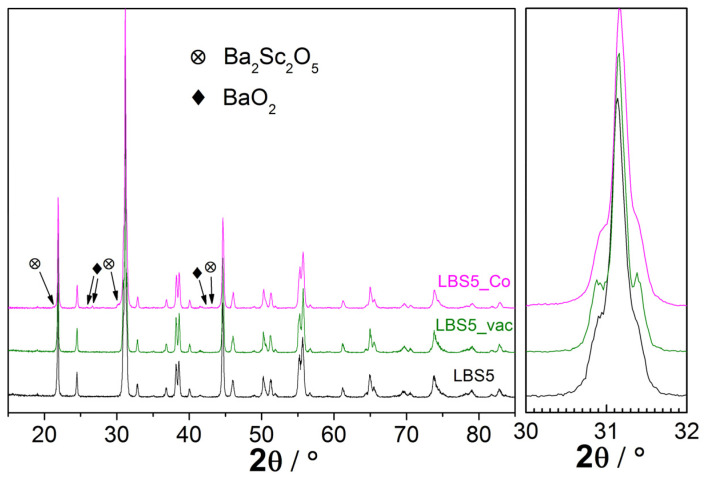
XRD patterns of La_0.95_Ba_0.05_ScO_3-δ_ samples obtained after standard sintering (1650 °C), vacuum sintering (1800 °C), and sintering with 0.5 wt% Co_3_O_4_ additive (1650 °C).

Figure 6 shows the XRD patterns of La_0.95_Ba_0.05_ScO_3-δ_ crushed ceramics: after standard sintering at 1650 °C, vacuum sintering at 1800 °C and sintering at 1650 °C with 0.5 wt% Co_3_O_4_ sintering additive at 1650 °C. The perovskite-like structure was confirmed in all cases, and as Table 1 shows, the unit cell parameters for all samples are very similar. In the case of the La_0.95_Ba_0.05_ScO_3-δ_ + 0.5 wt% Co_3_O_4_ sample, there are additional XRD peaks that may correspond to Ba_2_Sc_2_O_5_ [49] and peroxide BaO_2_ impurities.

**Figure 7:**
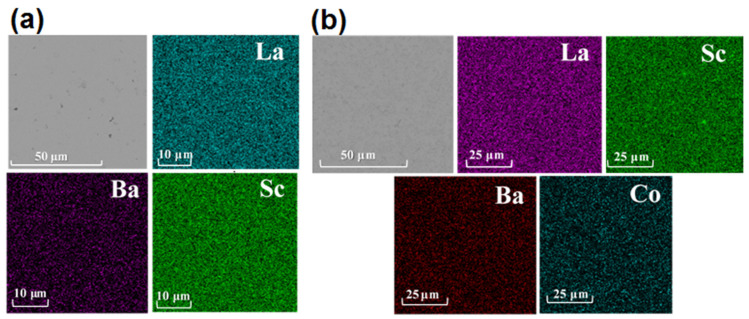
SEM images (BSE mode) of cross-sections for La_0.95_Ba_0.05_ScO_3-δ_ after vacuum sintering (**a**) and La_0.95_Ba_0.05_ScO_3-δ_ + 0.5 wt% Co_3_O_4_ (**b**) with EDX maps of cation distribution.

SEM images of cross-sections in Figure 7 demonstrate the absence of closed image pores in the volume of the ceramics. The results of EDX analysis show a homogeneous distribution of La, Ba, and Sc in the vacuum sintered sample (Figure 7a). At the same time, for La_0.95_Ba_0.05_ScO_3-δ_ + 0.5 wt% Co_3_O_4_, a small number of regions with an increased Sc concentration are noted (Figure 7b), which may correspond to Sc_2_O_3_. We do not detect areas enriched in Ba, indicating a uniform distribution of Ba in terms of EDX beam size. As seen from Table 2, the quantitative ratio of cations is in good agreement with the nominal compositions of the samples, although a slight deficit in the sum of La and Ba relative to Sc is observed. In this case, the amount of Ba relative to La is in good agreement with the given one and is equal to 0.05; therefore, in this case, the evaporation of Ba during synthesis does not occur even after high-temperature annealing in a vacuum at 1800 °C. Most likely, the use of sacrificial powder led to this good result.

**Figure 8:**
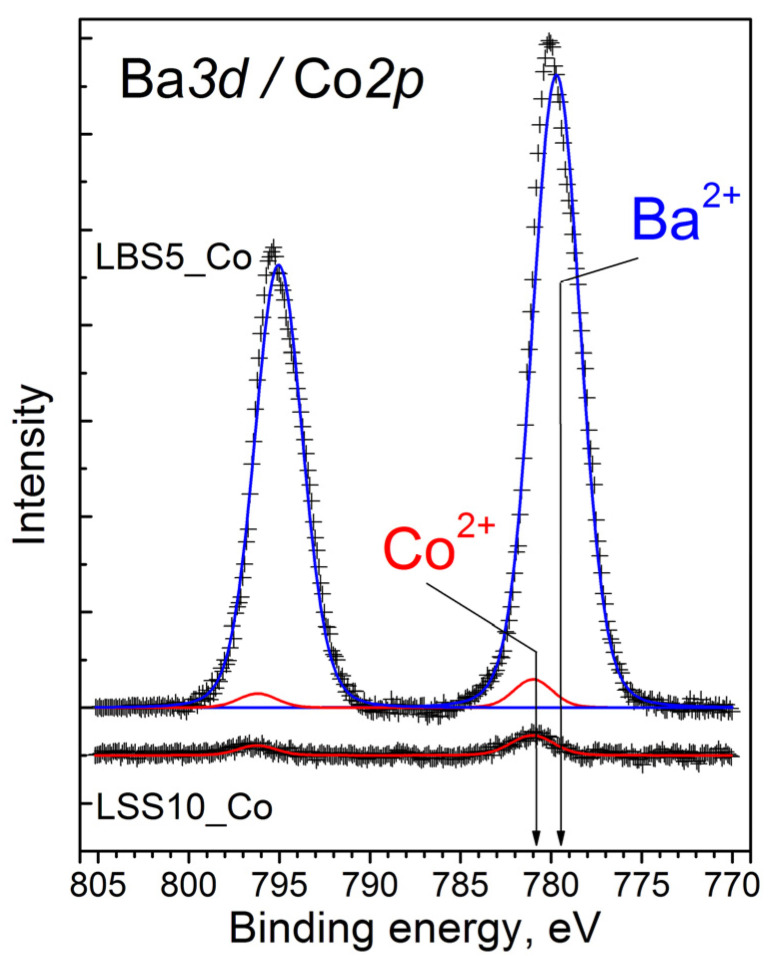
Ba*3d* (blue line) and Co*2p* (red line) core-levels spectra of La_0.95_Ba_0.05_ScO_3-δ_ + 0.5 wt% Co_3_O_4_ and La_0.9_Sr_0.1_ScO_3-δ_ + 0.5 wt% Co_3_O_4_ samples.

Figure 8 shows the Ba*3d* and Co*2p* core-level spectra of the La_0.95_Ba_0.05_ScO_3-δ_ + 0.5 wt% Co_3_O_4_ sample. The Ba*3d* spectra split into the Ba*3d_5/2_* and Ba*3d_3/2_* components (the spin-orbital splitting is 15.3 eV) due to the spin–orbit interaction. The Ba*3d_5/2_* binding energy is 780.0 eV, which corresponds to the Ba^2+^ state [50,51]. The Ba*3d* and Co*2p* spectra overlap substantially. For comparison, we present the data for the La_0.9_Sr_0.1_ScO_3-δ_ + 0.5 wt.% Co_3_O_4_ sample, which was studied in detail elsewhere [8]. The Co*2p* spectra are the Co*2p_3/2_*–Co*2p_1/2_* doublet. Cobalt in the Co^2+^ state is characterized by the Co*2p_3/2_* binding energies in the range 780.0–782.0 eV, as well as the presence of an intense (up to 20% of the main peak) ‘shake-up’ satellite in the 786–787 eV region [52,53,54]. As seen in Figure 8, the Co*2p_3/2_* is a symmetric peak at 781.0 eV; thus, the Co*2p* spectrum for La_0.9_Sr_0.1_ScO_3-δ_ + 0.5 wt% Co_3_O_4_ contains only one Co^2+^ component with a small intensity. This indicates that Co atoms diffused into the bulk of the grains. We expect a similar behavior for the La_0.95_Ba_0.05_ScO_3-δ_ + 0.5 wt% Co_3_O_4_ sample, which allows for a good fitting of the XPS spectrum as a sum of two components: Ba^2+^ and Co^2+^ (Figure 8). Elemental analysis indicates a threefold excess of Ba ([Ba]/[La] = 0.16) and slight excess of La ([La]/[Sc] = 1.2) on the surface of the sample.

### 3.4. Thermal Expansion

**Figure 9:**
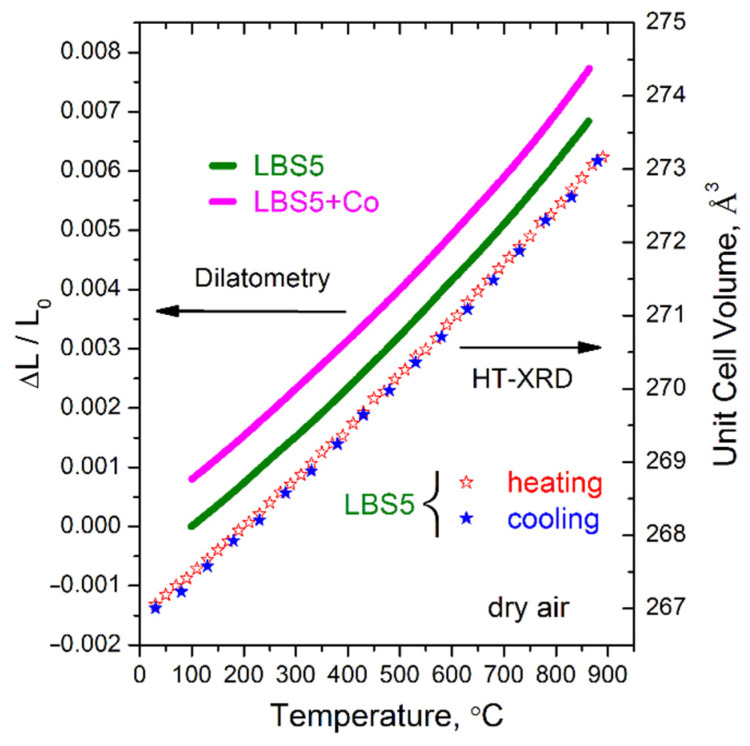
Thermal expansion of La_0.95_Ba_0.05_ScO_3-δ_ and La_0.95_Ba_0.05_ScO_3-δ_ + 0.5 wt% Co_3_O_4_ ceramic samples in dry air according to dilatometry and HT-XRD.

As shown in Figure 9, the linear dependence of thermal expansion in a dry air atmosphere according to dilatometry and HT-XRD data indicates the absence of structural transitions. Kim et al. [15] mentioned the formation of a mixture of orthorhombic and cubic phases for La_1-x_Ba_x_ScO_3-δ_ with x ≥ 0.1, while Kendrick et al. [31] definitely indicated an orthorhombic structure for La_0.73_Ba_0.27_ScO_3-δ_. For La_0.95_Ba_0.05_ScO_3-δ_, the orthorhombic structure with the *Pnma* space group is retained up to 900 °C. Dilatometric results show linear expansion of both La_0.95_Ba_0.05_ScO_3-δ_ and La_0.95_Ba_0.05_ScO_3-δ_ + 0.5 wt% Co_3_O_4_ ceramic samples with thermal expansion coefficient (TEC) values corresponding to 8.9 10^−6^ K^−1^, which is close to the values for Ca- and Sr-doped LaScO_3_ [20,21].

### 3.5. Water Uptake

**Figure 10:**
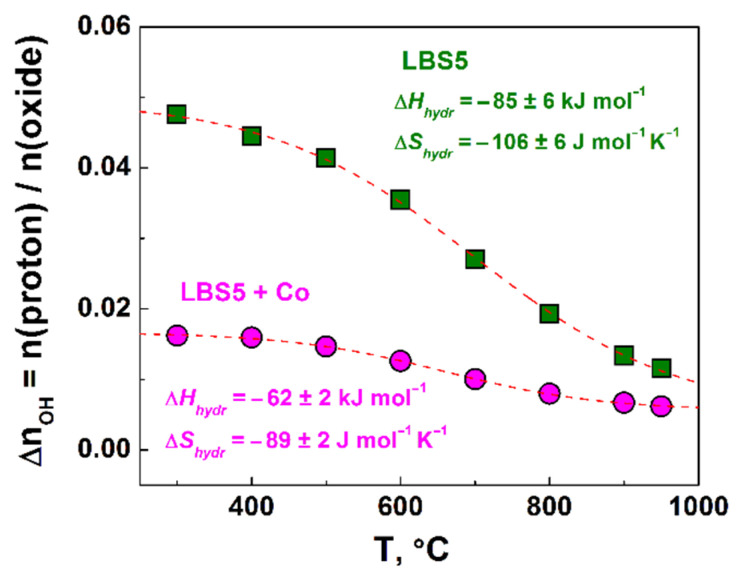
Proton concentrations in La_0.95_Ba_0.05_ScO_3-δ_ and La_0.95_Ba_0.05_ScO_3-δ_ + 0.5 wt% Co_3_O_4_ samples.

According to Equations (1) and (2), the value of maximal water uptake (*x_ef_*, saturation limit) has to be equal to the Ba acceptor dopant concentration $n_{OH}$ = 0.05 for the investigated La_0.95_Ba_0.05_ScO_3-δ_ sample. Figure 10 demonstrates the temperature dependence of the proton concentration in La_0.95_Ba_0.05_ScO_3-δ_ and La_0.95_Ba_0.05_ScO_3-δ_ + 0.5 wt% Co_3_O_4_ crushed ceramic samples, which were obtained by TGA. In the case of La_0.95_Ba_0.05_ScO_3-δ_, the proton uptake saturation limit *x_ef_* closely approaches the nominal Ba concentration. The calculated thermodynamic parameters of hydration Δ*H_hydr_* (standard enthalpy) and Δ*S_hydr_* (standard entropy) for La_0.95_Ba_0.05_ScO_3-δ_ were −85 ± 6 kJ mol^−1^ and −106 ± 6 J mol^−1^ K^−1^, respectively. These values are more positive than the values of Δ*H_hydr_* and Δ*S_hydr_* for Ca-doped [21] and Sr-doped [19,23] LaScO_3_ at the same concentration of the acceptor dopant. It is found that the proton concentrations for the La_0.95_Ba_0.05_ScO_3-δ_ + 0.5 wt% Co_3_O_4_ sample were significantly below the nominal acceptor concentration. Both Δ*H_hydr_* = −62 ± 2 kJ mol^−1^ and Δ*S_hydr_* = −89 ± 2 J mol^−1^ K^−1^ thermodynamic parameters became even more positive for La_0.95_Ba_0.05_ScO_3-δ_ + 0.5 wt% Co_3_O_4_. Thus, the results clearly indicate a negative effect of Co_3_O_4_ additive on water uptake, both on the concentration of protons and on the hydration thermodynamics.

### 3.6. Conductivity

#### 3.6.1. Total Conductivity

**Figure 11:**
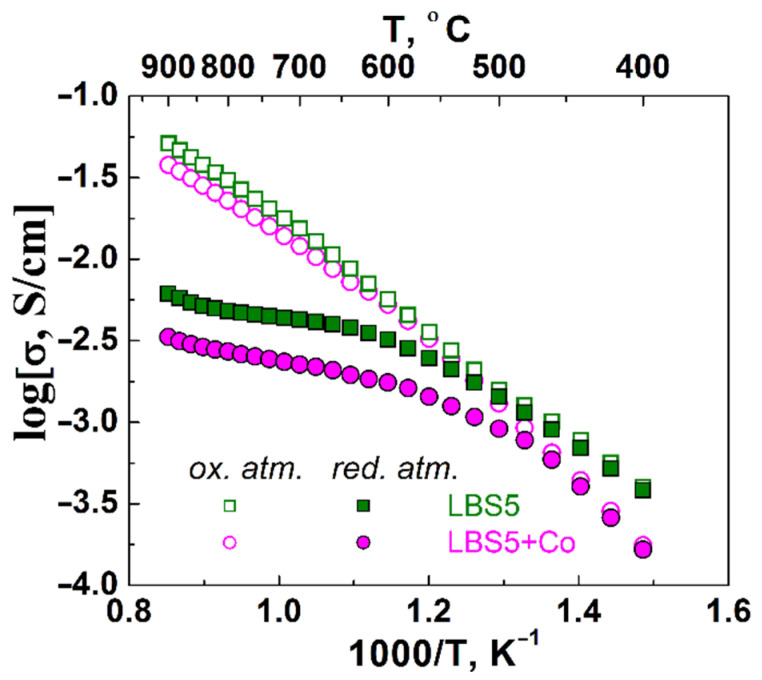
Temperature dependencies of electrical conductivity of La_0.95_Ba_0.05_ScO_3-δ_ and La_0.95_Ba_0.05_ScO_3-δ_ + 0.5 wt% Co_3_O_4_ ceramics in wet oxidizing and reducing atmospheres.

Figure 11 shows the temperature dependencies of the total conductivity of the La_0.95_Ba_0.05_ScO_3-δ_ and La_0.95_Ba_0.05_ScO_3-δ_ + 0.5 wt% Co_3_O_4_ samples in oxidizing and reducing atmospheres at *p*H_2_O = 2.8 kPa, measured using the 4-probe DC method. The dependencies in a reducing atmosphere demonstrate a clear inflection caused by the superposition of two types of ion transport: proton and oxygen ion. In an oxidizing atmosphere, they have an almost linear form, which is the result of the three charge carriers’ contribution (protons, oxygen ions, and holes). Holes are formed according to the oxidation reaction:

(7) $$\frac{1}{2}O_{2}+V_{O}^{••}=O_{O}^{\times }+2h^{•}\;$$

The predominance of hole conductivity at high temperatures leads to the linearization of the temperature dependence of the total conductivity. The total conductivity values of La_0.95_Ba_0.05_ScO_3-δ_ are higher than those of La_0.95_Ba_0.05_ScO_3-δ_ + 0.5 wt% Co_3_O_4_, primarily due to the reduced concentration of defects in the latter. The difference in the relative density of the La_0.95_Ba_0.05_ScO_3-δ_ (98%) and La_0.95_Ba_0.05_ScO_3-δ_ + 0.5 wt% Co_3_O_4_ (93%) materials should also not be ruled out, although it is not as significant as might be expected.

#### 3.6.2. Proton, Oxygen Ion, and Hole Partial Conductivities

**Figure 12:**
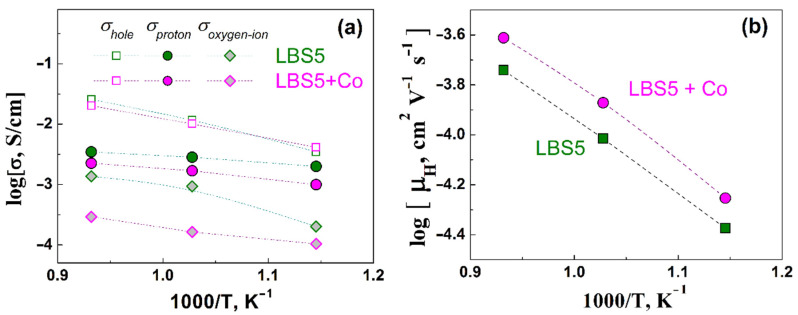
Temperature dependences of the proton, hole, and oxygen ion partial conductivities (**a**) and proton mobilities (**b**) of La_0.95_Ba_0.05_ScO_3-δ_ and La_0.95_Ba_0.05_ScO_3-δ_ + 0.5 wt% Co_3_O_4_ samples.

Figure 12a shows the temperature dependences of the partial hole, oxygen ion, and proton conductivity in a humid oxidizing atmosphere in the temperature range 800–600 °C. The hole conductivity dominates over oxygen ion and proton conductivity, which is associated with the high mobility of holes, although their concentration is rather low. The hole conductivity of La_0.95_Ba_0.05_ScO_3-δ_ and La_0.95_Ba_0.05_ScO_3-δ_ + 0.5 wt% Co_3_O_4_ is comparable, although the activation energy for La_0.95_Ba_0.05_ScO_3-δ_ + 0.5 wt% Co_3_O_4_ (~0.72 eV) is lower compared to that of La_0.95_Ba_0.05_ScO_3-δ_ without sintering additive (~0.89 eV). In contrast to the hole conductivity, the presence of a Co_3_O_4_ additive leads to a significant decrease in proton and oxygen ion conductivity. The proton mobility was calculated using the formula:

(8) $$\mu_{H}=\frac{\sigma_{H}}{e\cdot n_{H}}\;,$$

where $\sigma_{H}$ is the proton conductivity, $n_{H}$ is the proton concentration, and *e* is the elementary charge. As Figure 12b shows, with the introduction of the Co_3_O_4_ additive, the proton mobility increases. The activation energy (E_a_) of the proton mobility is very close, 0.67 eV and 0.68 eV for La_0.95_Ba_0.05_ScO_3-δ_ and La_0.95_Ba_0.05_ScO_3-δ_ + 0.5 wt% Co_3_O_4_, respectively.

#### 3.6.3. Bulk and GB Conductivity

**Figure 13:**
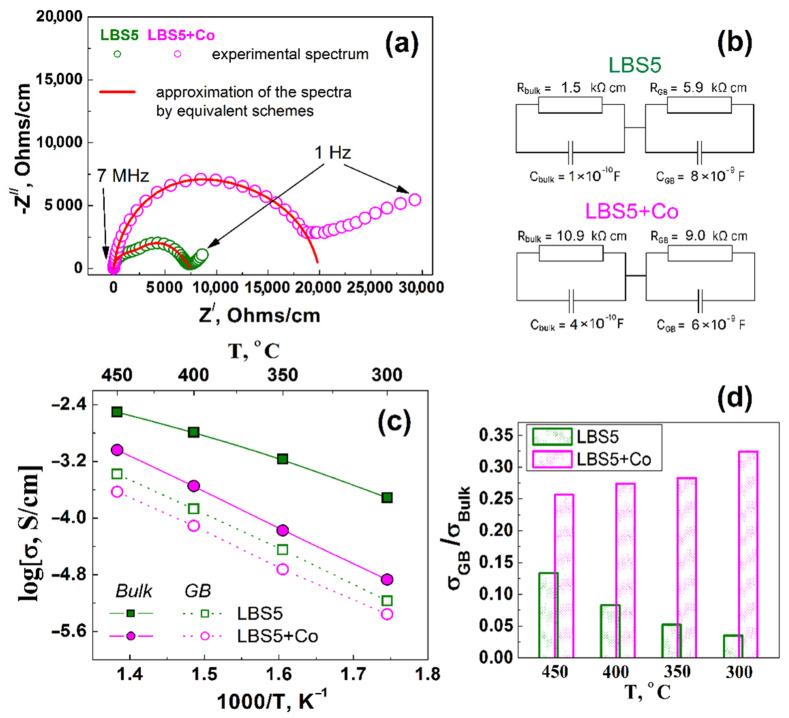
EIS results for La_0.95_Ba_0.05_ScO_3-δ_ and La_0.95_Ba_0.05_ScO_3-δ_ + 0.5 wt% Co_3_O_4_ ceramics: (**a**) impedance spectra at 350 °C, (**b**) equivalent schemes, (**c**) temperature dependences of the bulk and GB conductivities, (**d**) σ_GB_/σ_Bulk_ ratios.

The conductivity of the bulk and grain boundaries (GB) of the La_0.95_Ba_0.05_ScO_3-δ_ and La_0.95_Ba_0.05_ScO_3-δ_ + 0.5 wt% Co_3_O_4_ ceramics was measured in the temperature range 300–450 °C in a humidified oxidizing atmosphere using EIS. Figure 13a shows the typical impedance spectra of the samples studied, and Figure 13b shows the corresponding equivalent circuits. Figure 13c shows the temperature dependences of bulk and GB conductivity, and in Figure 13d, they are shown as σ_GB_/σ_Bulk_ ratios. The difference between the GB and bulk conductivities of La_0.95_Ba_0.05_ScO_3-δ_ increases with decreased in temperature, resulting in the significant impact of the GB contribution. The bulk conductivity of the La_0.95_Ba_0.05_ScO_3-δ_ + 0.5 wt% Co_3_O_4_ sample is an order of magnitude lower than that of La_0.95_Ba_0.05_ScO_3-δ_. In terms of the σ_GB_/σ_Bulk_ ratios, the contribution of GB conductivity is larger in the La_0.95_Ba_0.05_ScO_3-δ_ + 0.5 wt% Co_3_O_4_ sample. As shown in Table 3, the effective activation energy (E_a_) for the bulk conductivity of La_0.95_Ba_0.05_ScO_3-δ_ + 0.5 wt% Co_3_O_4_ have higher values compared to La_0.95_Ba_0.05_ScO_3-δ_, which indicates the negative effect of the sintering additive on the transfer of charge carriers. At the same time, the E_a_ values for GB conductivity are comparable.

## 4. Discussion

Despite the fact that the homogeneity region of La_1-x_Ba_x_ScO_3-δ_ solid solutions is located in a wide range up to x ~ 0.4 [15,31], an increase in the Ba content already leads to a significant decrease in the ceramic densityin the x ~ 0–0.1 concentration range (Figure 2). We considered two strategies for obtaining dense La_0.95_Ba_0.05_ScO_3-δ_ ceramics: increasing the sintering temperature and introducing a sintering additive. It is understood that the sintering additive should promote both sintering due to a decrease in the material’s melting temperature at the GBs and the incorporation of the dopant into the crystal lattice due to a decrease in carbonization of the Ba in the grains surface.

The production of dense La_0.95_Ba_0.05_ScO_3-δ_ ceramics without a sintering additive becomes possible only by high-temperature sintering in a vacuum at 1800 °C. In this case, large grains with a size of 7.1 ± 1.2 μm are formed (Figure 4c). The phase and chemical composition of the material correspond to the nominal ones thanks to the use of sacrificial coating powder, which prevents the evaporation of components, primarily Ba. In addition, no structural transitions were found in La_0.95_Ba_0.05_ScO_3-δ_ up to 900 °C, according to HT-XRD analysis (Figure 9).

In the case of the Co_3_O_4_ sintering additive in La_0.95_Ba_0.05_ScO_3-δ_, the XRD and SEM results give us complex data. As SEM-EDX analysis in Figure 7b shows, the addition of 0.5 wt% Co_3_O_4_ in La_0.95_Ba_0.05_ScO_3-δ_ results in the release of a small amount of Sc_2_O_3_, which is not detected by XRD [31]. The results of XRD analysis indicate partial decomposition of the material with the formation of Ba_2_Sc_2_O_5_ and BaO_2_ phases with a total mass fraction of about 1.5 wt%. Similarly, the formation of impurity phases in Ba(Ce,Zr,Y)O_3-δ_ with Fe_2_O_3_ and NiO sintering additives was observed in a number of works [36,55,56,57]. EDX analysis of the cross sections does not detect these phases, which may be due to their small size and uniform distribution. A similar situation was obtained by Shakel et al. [56] for BaZr_0.8_Y_0.2_O_3-δ_ with NiO sintering additive, indicating that even transmission electron microscopy does not detect impurity phases in the form of individual grains. The XPS results in Figure 8 indicate that the Co content on the grain surface of the La_0.95_Ba_0.05_ScO_3-δ_ + 0.5 wt% Co_3_O_4_ material is negligible, confirming that Co atoms diffuse into the bulk of the grains. In addition, a noticeable part of Ba is displaced to the surface of the grains, which reduces its concentration in the grain volume. Thus, this should lead to a decrease in the concentration of oxygen vacancies formed when La is replaced by Ba in accordance with Equation (1). However, the actual concentration of Ba in the volume of the La_0.95_Ba_0.05_ScO_3-δ_ + 0.5 wt% Co_3_O_4_ sample grains corresponds to 0.048 according to EXD of the ceramics’ cross-sections (Table 2), which indicates a small contribution of Ba segregation towards the GBs.

A small amount of 0.5 wt% Co_3_O_4_ additive cannot explain the significant decrease in the ionic conductivity of the material, as well as the water uptake, only due to the binding of Ba to the Co. In addition, an increase in the Ba dopant concentration at a constant of Co_3_O_4_ sintering additive concentration leads to an increase in the precipitation of the Sc_2_O_3_ phase (Figure S3). The general stoichiometry of La_0.95_Ba_0.05_ScO_3-δ_ is obviously violated when the sintering additive is introduced, which leads to partial decomposition of the material, in particular, the formation of Ba-enriched phases. Recently, Huang et al. [35] listed a number of effects from the introduction of a NiO sintering additive into BaZrO_3_-type materials. Violation of the cationic stoichiometry can cause the formation of cationic vacancies. Being a charged defect, cation vacancies can form associates with oxygen vacancies, which significantly reduces their mobility [56,58]. Additional trapping of oxygen vacancies is possible by Co atoms, which are embedded in Sc positions. Recent molecular dynamics modeling confirms the possibility of the association of Co with oxygen vacancies in La_0.9_Sr_0.1_Sc_1-y_Co_y_O_3-δ_ [59]. Thus, some of the oxygen vacancies that form in accordance with Equation (1) and are directly related to the Ba dopant concentration may be associated with other defects and not participate in the oxygen ionic conductivity or water uptake reaction (Equation (2)).

The reduced oxygen vacancy content obviously reduces the saturation limit during hydration (*x*_ef_). As Figure 9 shows, *x*_ef_ noticeably decreases from 0.048 for the sample without the sintering additive to 0.018 for the sample with the sintering additive. Apparently, it leads to a significant deterioration in proton transport (Figure 12a). It is fair to assume that Co atoms are partially included in the Sc site of the La_0.95_Ba_0.05_ScO_3-δ_ crystal lattice, displacing part of the Sc atoms and forming the La_0.95_Ba_0.05_Sc_1-y_Co_y_O_3-δ_ phase. Since Co can have an oxidation state of either +2 or +3, it can serve as a source of additional holes. The transfer of electron holes to oxygen atoms will lower the ‘basicity’ of oxygen sites [60]; in other words, it reduces the effective negative charge on oxygen atoms. This assumption is directly confirmed by the fact that the value of the standard enthalpy of hydration for La_0.95_Ba_0.05_ScO_3-δ_ + 0.5 wt% Co_3_O_4_ (Δ*H_hydr_* = −62 kJ mol^−1^) becomes more positive compared to La_0.95_Ba_0.05_ScO_3-δ_ (Δ*H_hydr_* = −85 kJ mol^−1^), and thus, hydration becomes less favorable. This effect also leads to an increase in the mobility of protons in La_0.95_Ba_0.05_ScO_3-δ_ + 0.5 wt% Co_3_O_4_ compared to La_0.95_Ba_0.05_ScO_3-δ_ (Figure 12b). On the other hand, Kim et al. [61] mention a decrease in the mobility of protons when Ni is introduced into BaZr_0.8_Y_0.2_O_3-δ_, which can be caused by the trapping of protons near Ni atoms.

The introduction of Co in La_0.95_Ba_0.05_ScO_3-δ_ leads to a slight decrease in the negative contribution of the GB resistance (Figure 13d). As well, the effective activation energy of GB conductivity for La_0.95_Ba_0.05_ScO_3-δ_ (0.98 eV) is higher than for La_0.95_Ba_0.05_ScO_3-δ_ + 0.5 wt.\% Co_3_O_4_ (0.95 eV). The positive effect of the introduction of sintering additives was previously also mentioned by the authors [62,63,64] for Ba(Ce,Zr)O_3_-based materials with NiO, CuO, ZnO, Co_3_O_4_ sintering additives and is associated simply with an improvement in the contact between grains. Some authors [65,66] explained the decrease in the GB resistance by pointing to an increase in the concentration of charge carriers in the space charge layers and with lower potential transfer barrier. Most likely, this is facilitated by an increase in the transport numbers of holes (Figure 12a). The negative effect on the specific GB conductivity of Ba(Zr,Y)O_3_ with the NiO additive [56,67] is associated with the blocking of ion transfers through the grain boundaries, whereas in our case, no such effect is observed.

## 5. Conclusions

The Ba dopant in La_1-x_Ba_x_ScO_3-δ_ causes deterioration of ceramic sintering already at low concentrations of x = 0.025–0.1, leading to a sharp decrease in the ceramic density up to 76% after sintering at 1650 °C for 5 h, as well as a decrease in the average grain size from 0.97 to 0.34 μm. Two strategies have been applied for obtaining dense ceramics: high processing temperatures and the introduction of a Co_3_O_4_ sintering additive. The use of 0.5 wt% Co_3_O_4_ sintering additive, despite its low concentration, caused a number of negative consequences. The set of methods used indicates a violation of the overall stoichiometry of the material and the formation of impurity phases: Ba_2_Sc_2_O_5_, BaO_2_, and Sc_2_O_3_. A significant deterioration in the ionic conductivity of the material may indicate an association of cationic and oxygen vacancies, which excludes the latter from oxygen transport and water uptake. The incorporation of Co into Sc positions causes a weakening of the binding energy of oxygen with a proton due to the transfer of electron holes from Co to oxygen. This causes an increase in the enthalpy of the hydration reaction and proton mobility. Thus, the use of sintering additives for LaScO_3_-based materials can lead to a number of negative consequences, since it significantly affects the actual concentration of the dopant and the cationic stoichiometry of the material. Highly dense ceramics (98%) can only be obtained by high-temperature sintering at 1800 °C, which also leads to a significant increase in the average grain size to 7.08 μm. The accordance with the concentration of elements of the nominal composition is achieved by covering the materials with a protective powder of a similar composition, which, in particular, prevents Ba evaporation. The obtained electrolyte membranes demonstrate high water uptake and proton conductivity.

## Figures and Tables

**Table 1:** Unit cell parameters of La_0.95_Ba_0.05_ScO_3-δ_ samples sintered in various ways.

Sample	A, Å	B, Å	C, Å	Volume, Å^3^
La_0.95_Ba_0.05_ScO_3-δ_ standard sintering	5.780	8.110	5.697	267.1
La_0.95_Ba_0.05_ScO_3-δ_ vacuum 1800 °C	5.784	8.105	5.692	266.9
La_0.95_Ba_0.05_ScO_3-δ_ + 0.5 wt% Co_3_O_4_	5.778	8.106	5.693	266.7

**Table 2:** Element contents (at.% ± 0.05) in the ceramics cross-sections according to EDX analysis.

Sample	La	Ba	Sc	Co	[Ba]/[La]
La_0.95_Ba_0.05_ScO_3-δ_ vacuum 1800 °C	47.25	2.42	50.33	-	0.05
La_0.95_Ba_0.05_ScO_3-δ_ + 0.5 wt% Co_3_O_4_	46.87	2.27	49.98	0.88	0.048

**Table 3:** Effective activation energies of the bulk and GB conductivity of La_0.95_Ba_0.05_ScO_3-δ_ and La_0.95_Ba_0.05_ScO_3-δ_ + 0.5 wt% Co_3_O_4_ ceramics.

Conductivity	LBS	LBS + Co
Bulk	0.62 eV	0.89 eV
GB	0.98 eV	0.95 eV

## Data Availability

Not applicable.

## Supplementary Materials

The following supporting information can be downloaded at: https://www.mdpi.com/article/10.3390/membranes12111084/s1, Figure S1: Conductivity of ceramics as a function of (*p*O_2_)^1/4^ under wet (*p*H_2_O = 2.8 kPa) and dry (*p*H_2_O = 0.1 kPa) conditions; Figure S2: La*3d* and Sc*2p* core-level spectra La_0.95_Ba_0.05_ScO_3-δ_ + 0.5 wt% Co_3_O_4_ and La_0.9_Sr_0.1_ScO_3-δ_ + 0.5 wt% Co_3_O_4_ samples; Figure S3: SEM-images of the surface (a) and cross-sections (b) of La_0.925_Ba_0.075_ScO_3-δ_ + 0.5 wt% Co_3_O_4_ and La_0.9_Ba_0.1_ScO_3-δ_ + 0.5 wt% Co_3_O_4_ ceramics with EDX maps of cations distribution.
