# Supplementary material for: Densification and Proton Conductivity of La1-xBaxScO3-δ Electrolyte Membranes
Source: Membranes (Basel). 2022 Oct 31;12(11):1084. doi: 10.3390/membranes12111084 (PMC9698234; doi:10.3390/membranes12111084)
Supplement: Supplementary file 1 [file membranes-12-01084-s001.zip › membranes-1978321-supplementary-done.pdf]

membranes

Supplementary Information

Densification and Proton Conductivity of $La_{1-x}Ba_xScO_{3-\delta}$ Electrolyte Membranes

Alyona Lesnichyova [1,2], Semyon Belyakov [3], Anna Stroeva [4], Sofia Petrova [5], Vasiliy Kaichev [6] and Anton Kuzmin [1,4,*]

1 Laboratory of Solid-State Chemistry, Institute of Solid-State Chemistry and Mechanochemistry SB RAS, Novosibirsk 630128, Russia
2 NANOTECH Centre, Ural Federal University, Yekaterinburg 620075, Russia
3 Laboratory of Electrochemical Materials Science, Institute of High-Temperature Electrochemistry UB RAS, Yekaterinburg 620137, Russia
4 Department of Technology of Inorganic Materials and Electrochemical Production, Vyatka State University, Kirov 610000, Russia
5 Laboratory of High-Entropy Alloys, Institute of Metallurgy UB RAS, Yekaterinburg 620016, Russia
6 Department of Catalysis Research, Boreskov Institute of Catalysis SB RAS, Novosibirsk 630090, Russia
* Correspondence: a.v.kuzmin@yandex.ru

pO_2 – dependencies of conductivity

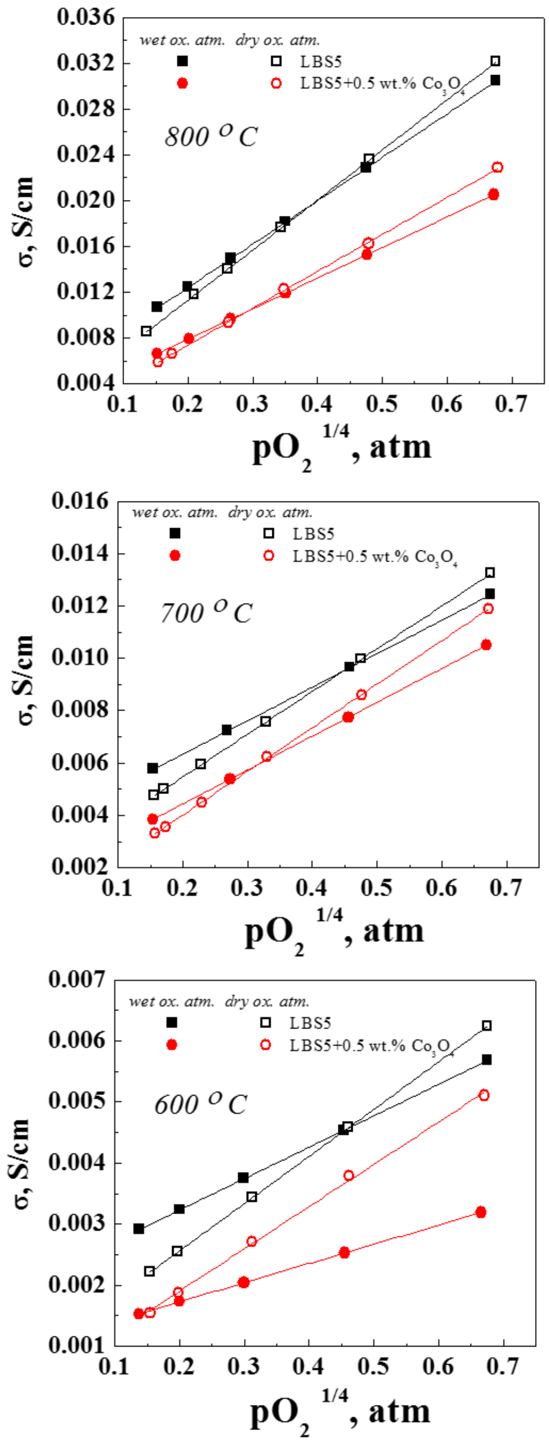

Figure S1. Conductivity of ceramics as a function of (pO_2)$^{1/4}$ under wet (pH_2O = 2.8 kPa) and dry (pH_2O = 0.1 kPa) conditions.

XPS analysis

The La3*d* level splits into two sublevels as a result of the spin-orbit interaction, which leads to the appearance of a doublet $La3d_{5/2}$ - $La3d_{3/2}$ (~16.8 eV). Each La^{3+} component in the La3*d* spectra is accompanied by intense 'shake-up' lines of the satellites. The main $La3d_{5/2}$ peak for La_2O_3 is located in the region of 833.2-834.0 eV. For lanthanum carbonate $La_2(CO_3)_3$, slightly higher binding energies of $La3d_{5/2}$ are observed in the range of 835.0-835.5 eV, and for lanthanum hydroxides LaOOH and $La(OH)_3$ it is about 834.8 eV. In our case, La^{3+} is in the form of La_2O_3 or $LaSrO_x$.

The Sc2*p* level splits into two sublevels $Sc2p_{3/2}$ and $Sc2p_{1/2}$ (4.46 eV) due to the spin-orbit interaction. In the literature, the binding energy values of $Sc2p_{3/2}$ for Sc^{3+} are given in the range 401.7–402.5 eV, and in ScOOH it is 403.0 eV. The Sc2*p* spectra are described by a single doublet with a $Sc2p_{3/2}$ binding energy 402.0 eV (taking into account the correction of the binding energy by +1.9 eV). Thus, Sc^{3+} is in the form of Sc_2O_3 or $ScSrO_x$.

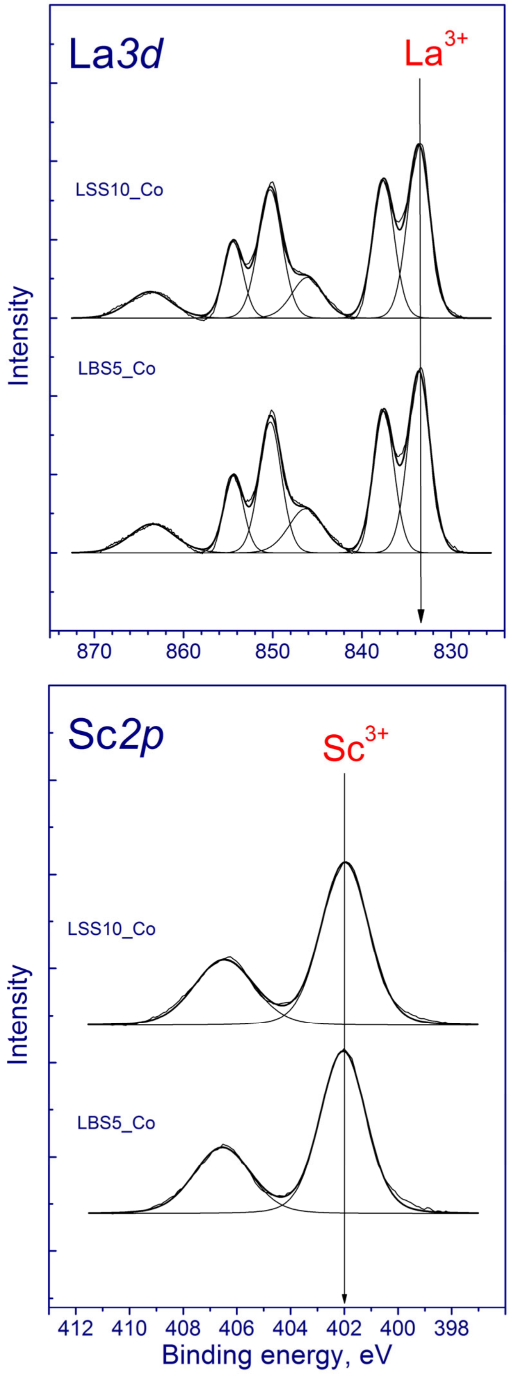

Figure S2. La*3d* and Sc*2p* core-level spectra $La_{0.95}Ba_{0.05}ScO_{3-\delta}$ + 0.5 wt% Co_3O_4 and $La_{0.9}Sr_{0.1}ScO_{3-\delta}$ + 0.5 wt% Co_3O_4 samples.

Phase equilibria (Ba-Co)

The Sc-enriched phase is formed predominantly on the surface of ceramic samples, while its concentration in the bulk of the samples is low.

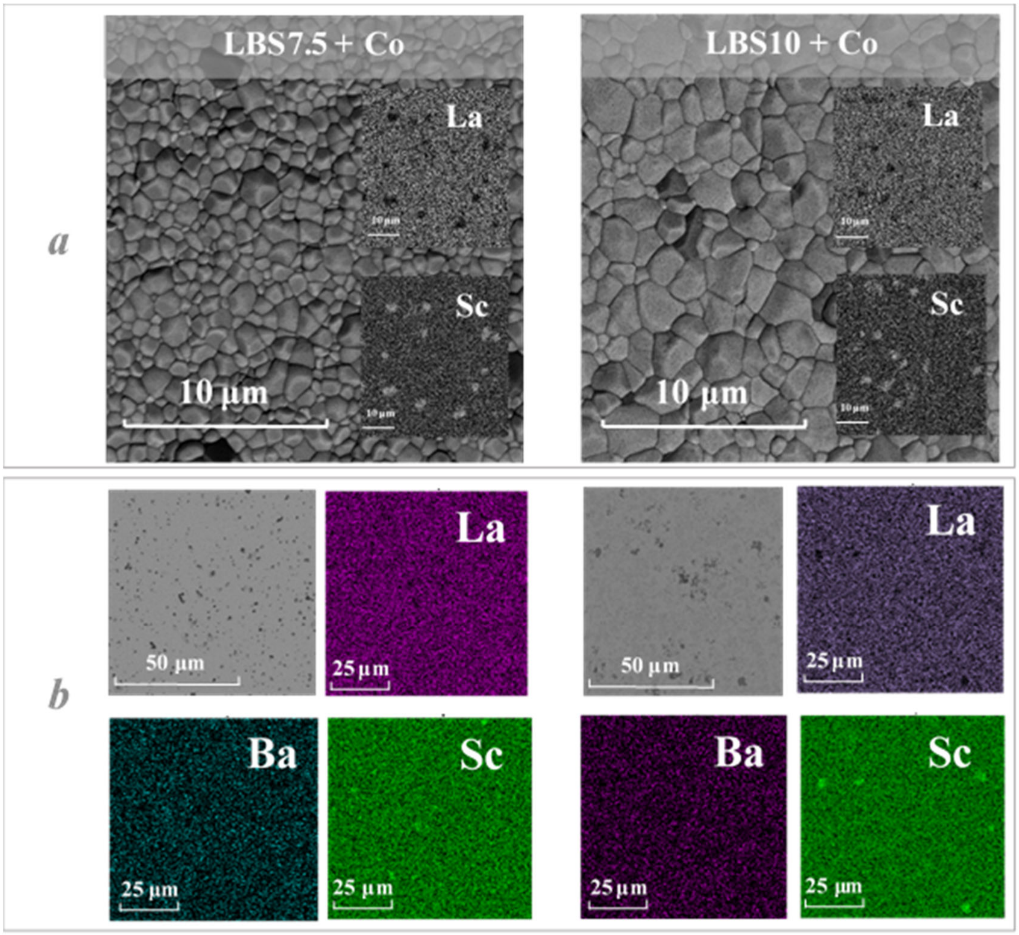

Figure S3. SEM-images of the surface (**a**) and cross-sections (**b**) of $La_{0.925}Ba_{0.075}ScO_{3-\delta}$ + 0.5 wt% Co_3O_4 and $La_{0.9}Ba_{0.1}ScO_{3-\delta}$ + 0.5 wt% Co_3O_4 ceramics with EDX maps of cations distribution.
